# Isolated Ventricular Noncompaction Syndrome in a Nigerian Male: Case Report and Review of the Literature

**DOI:** 10.4061/2010/539538

**Published:** 2010-07-15

**Authors:** Indrani S. Bhattacharya, Marc Dweck, Andrew Gardner, Mark Jones, Mark Francis

**Affiliations:** Cardiology Unit, Victoria Hospital, Kirkcaldy, Fife KY2 5AH, Scotland

## Abstract

Isolated ventricular non-compaction cardiomyopathy (IVNC) is a rare, morphologically distinct primary genetic cardiomyopathy, which is now gaining prominence as an important differential diagnosis in patients presenting with cardiac failure. We describe a case report of a Nigerian male with facial dysmorphism presenting with cardiac failure. This is followed by a review of the literature with focus on the diagnosis of this condition, which may be difficult especially in non-Caucasian populations.

## 1. Case

A 42-year-old Nigerian man presented with a three-month history of worsening dyspnoea, orthopnoea, and paroxysmal nocturnal dyspnoea. He had no associated chest pain. He had no significant past medical history, was a nonsmoker, and drank minimal alcohol. He had been living in Scotland for several years and there was no recent history of foreign travel. There was no family history of cardiomyopathy or sudden death. 

**Figure 1:**
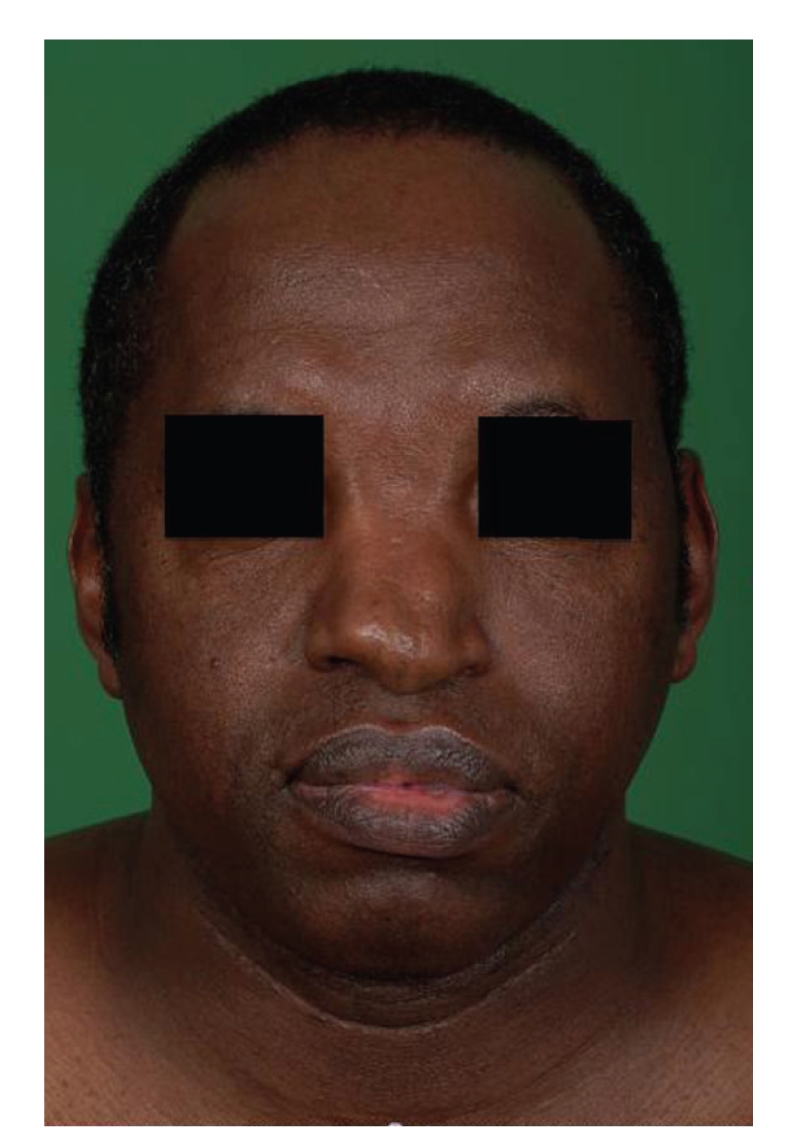
Facial appearance of our patient. Image shows a broad dorsum and asymmetrical tip of the nose with mild hypertelorism. This is likely to represent a congenital craniofacial deformity.

On examination he was afebrile with evidence of mild facial dysmorphism (Figure 1). He had a resting tachycardia with a blood pressure of 180/115. His JVP was raised, apex beat was impalpable, and he had a summation gallop with no murmurs. He had no ankle or sacral oedema. Auscultation of his chest revealed bibasal crepitations. 

**Figure 2:**
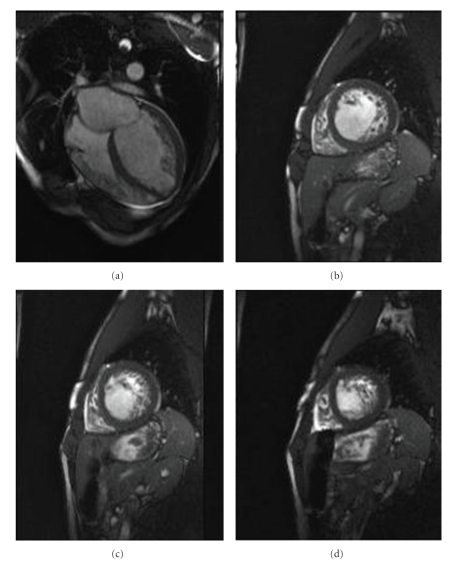
CMR images. Four chamber and short-axis views through the basal, mid-chamber, and apical segments in end diastole, demonstrating a 2-layered compacted and non-compacted myocardium. This is observed in the free wall of the left ventricle, particularly towards the apex although unusually the true apex is spared-. Differentiation from the papillary muscles (not shown) is made on the basis that this structure did not communicate with the mitral valve on the long axis.

**Figure 3:**
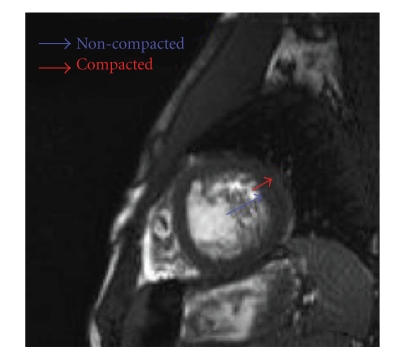
CMR measurements of the compacted (8 mm) and non-compacted (21 mm) layers of the myocardium.

He had a normal full blood count, urea, creatinine and electrolytes, liver function and thyroid function tests. He was HIV negative and his antinuclear factor was negative. His electrocardiogram showed a sinus tachycardia with voltage criteria for left ventricular hypertrophy. Troponin and CK levels were normal. His chest X-ray showed cardiomegaly with upper-lobe diversion. Echocardiographic images were suboptimal in quality but showed a dilated left ventricle, and severe global impairment of LV systolic function. Cardiac MRI (CMR) revealed similar findings but in addition demonstrated deep ventricular recesses and hypertrabeculation of the lateral wall extending down towards the apex (Figure 2). Unusually the true apex was not involved. The ratio of the compacted to noncompacted myocardium was 2.6 in diastole (Figure 3). Ejection fraction was 15% and there was no late gadolinium enhancement. 

In combination with his facial dysmorphism, these features were felt to be consistent with a diagnosis of LV noncompaction. Given the lack of anginal symptoms or risk factors, it was felt that coronary angiography was not necessary. Our patient did not describe any neurological symptoms. Furthermore complete examination of the nervous system and assessment of visual acuity were normal; therefore additional neurological investigations were not deemed necessary. 

Our patient was commenced on an ACE inhibitor, diuretic, beta blocker, and long-term anticoagulation. We advised him to contact his relatives back in Nigeria in order to arrange screening investigations. On followup, he has remained asymptomatic and has not required any further hospitalization. Holter monitoring has proved reassuring and repeat echocardiography suggested improvement in function, with the ejection fraction calculated at 45%. ICD insertion has therefore not been recommended at this time.

## 2. Discussion

Isolated ventricular non-compaction cardiomyopathy (IVNC) is a rare, morphologically distinct primary genetic cardiomyopathy, which was first described almost two decades ago. It is now gaining prominence as an important differential diagnosis in patients presenting with cardiac failure. It is characterized anatomically by prominent trabeculations in the ventricular wall and deep intertrabecular recesses which communicate with the main ventricular chamber [1]. This results in a two-layered structure of compacted and non-compacted myocardium, which allows differentiation from normal trabeculations, false tendons, and papillary muscles.

### 2.1. Aetiology

IVNC appears to be a genetically inherited condition that has been associated with many different gene mutations [2, 3]. Both X-linked and autosomal-dominant inheritances have been described in different families [4, 5]. As a result, first-degree relatives of patients diagnosed with IVNC require echocardiographic screening [1].

The exact cause of IVNC, however, remains controversial. The congenital theory suggests that it is due to failure in the compaction of the myocardium, which usually occurs between weeks five and eight of embryonic life [6]. This process begins in the septum and base of the heart, which might explain why the apex and lateral are most frequently involved in IVNC. However this hypothesis lacks proof and is largely based upon the similarities in appearances between the embryonic heart and IVNC. Other theories have been proposed which include dissection of the myocardium, frustrated attempts at hypertrophy, compensatory hypervascularisation, myocardial tearing due to dilatation and metabolic defects [7].

Nonisolated forms of non-compaction also exist which are associated with other forms of congenital heart disease including atrial septal defects, ventricular septal defects, aortic stenosis, pulmonary stenosis, tricuspid stenosis, and hypoplastic left ventricle. The aetiology of non-compaction in non-isolated forms may be different to IVNC [2]. 

### 2.2. Associated Clinical Disorders

Since the very early reports of non-compaction, an association has been noted with different forms of facial dysmorphism, particularly when the diagnosis is made in children [2, 8–10]. IVNC is also frequently associated with neuromuscular disorders such as Leber's hereditary optic neuropathy, Becker's muscular dystrophy, myotonic dystrophy, and metabolic myopathy [11]. One study suggested that 80% of patients with IVNC also had a neuromuscular disorder and neurological screening is therefore recommended in all patients [11].

### 2.3. Clinical Features and Management

Dyspnoea is the most common symptom alongside other manifestations of cardiac failure. Cardiac arrhythmias are also frequent and may be life threatening. The incidence of thromboembolic events is particularly high. It is postulated that they are the result of thrombus formation within the deep intra-trabecular recesses. However these events are often observed in the context of other prothrombotic factors such as atrial fibrillation and impaired systolic function [7]. In addition the incidence of pulmonary embolism is also relatively high [11], which suggests either involvement of the right ventricle or a more systemic tendency to thrombosis. Anticoagulation should be carefully considered [1] and, we believe, should be instigated in the context of atrial fibrillation, systolic impairment, or prior embolic events.

Otherwise management of IVNC focuses on conventional treatment of clinical manifestations. Patients with decreased ventricular systolic function should be treated with ACE inhibitors and beta blockers [12, 13]. At the end stages transplantation may be required [14]. Ambulatory ECG monitoring to assess for arrhythmias is appropriate for those with systolic dysfunction given the frequency of ventricular tachyarrhythmias and potential risk of sudden cardiac death [15]. Patients may require ICD insertion depending on these results, systolic function, and family history of sudden death [15]. 

Prognosis is variable; early studies suggested that it was poor [9] but more recent studies, which have included more subtle phenotypes, describe a much lower incidence of death stroke or ventricular arrhythmia [12]. Predictors of an increased mortality include increased age, neuromuscular disorders, cardiac failure, and left ventricular dilatation/dysfunction [16].

### 2.4. Diagnosis

Given the significant morbidity and mortality associated with IVNC, prompt and accurate recognition by cardiac imaging techniques is essential. Until recently isolated IVNC was thought to be extremely rare with a prevalence in adults of less than 0.3% [17]. However, there has been a marked increase in the diagnosis of IVNC, especially in asymptomatic patients. This has been attributed to the improvements in modern imaging techniques as well as routine screening of relatives of affected patients [18]. However, there are now concerns that IVNC may be overdiagnosed. 

Echocardiography is the first-line investigation for patients presenting with cardiac failure. Echocardiographic criteria for the diagnosis of IVNC have been established (Table 1), with the diagnosis being confirmed by a ratio of the maximal thickness of the non-compacted to compacted layers, of greater than 2 : 1, in systole, in the parasternal short-axis view. However these definitions are based on small studies and are likely to be overly sensitive: in one study 25% of all patients with LV systolic impairment referred to the heart failure clinic fulfilled criteria for IVNC [19]. 

Cardiac MRI (CMR) is an alternative imaging technique, which offers superior contrast between the myocardium and the blood pool. Diagnosis of IVNC can be made using CMR and demonstration of a non-compacted:compacted ratio >2.3, in end diastole, on long-axis views, with a sensitivity of 86% and a specificity of 99% [20]. In this study, the gold standard assessment against which CMR was tested was a clinical diagnosis of IVNC. The criteria for this clinical diagnosis ware demonstration of a two-layered myocardium in addition to: a documented, similar appearance in a first-degree relative, an associated neuromuscular disorder; regional wall motion abnormalities, or complications such as systemic emboli [20]. This highlights the difficulty in studying this condition whilst definitive diagnostic criteria are lacking. 

CMR studies have also led to the realization that a lesser degree of non-compaction may also be observed in normal ventricles [20]. This is corroborated by autopsy findings, which demonstrate that up to 70% of hearts display at least one prominent left ventricular trabeculation, with two or more present in 36% [21]. No patients had more than five, suggesting that the number of trabeculations may be a useful discriminatory finding [11]. 

Hypertrabeculation has also been reported in the context of other forms of cardiomyopathy, most commonly in hypertrophic cardiomyopathy and dilated cardiomyopathy. Interesting case reports have described families, in whom the same gene mutation has caused a dilated cardiomyopathy in some members and IVNC in others [22]. It may be, therefore, that hypertrabeculation in these cardiomyopathies exists on a spectrum, with IVNC at the extreme end.

### 2.5. Noncompaction in Different Ethnic Groups

Assessment of non-compaction is particularly difficult in black patients who display a greater degree of trabeculation of the ventricle than other groups. In one study 13% of healthy black control subjects fulfilled echo criteria for IVNC despite having no other features suggestive of a cardiomyopathy [19]. 

There is in fact growing evidence that the prevalence of IVNC differs in different ethnic groups. An Australian study compared rates of cardiomyopathy between indigenous and nonindigenous children and showed a much higher incidence of IVNC in the indigenous population [23], whilst a study based in North Africa showed that the incidence of IVNC amongst black Africans and Arabs was again high. Of relevance to our case, this study also described improvement in LV function in a subgroup of patients [24]. 

It is unclear whether these observations reflect a truly higher incidence of IVNC in these ethnic groups or are due to oversensitivity of the diagnostic criteria, which have been established in a predominantly Caucasian population.

### 2.6. Our Case

Our patient was a black African male, which made application of existing diagnostic criteria difficult. However, the degree of hypertrabeculation in combination with the established systolic impairment and facial dysmorphism was in our opinion sufficient to make a secure diagnosis of IVNC. It should be noted that it is unusual for the true apex not to be involved. However this has been described: Thuny et al. observed sparing of the true apex by CMR in 1 of the 16 patients studied. All 16 patients, however, had involvement of the lateral apical segment, which was the most clearly affected segment in our patient [25].

## 3. Conclusion

In summary, the diagnosis of IVNC is currently made challenging because of the heterogeneity in the degree of trabeculation observed in patients with normal ventricles and other forms of cardiomyopathy. Current diagnostic criteria are based upon studies involving only a small number of patients and there are concerns that they are leading to the overdiagnosis of this condition. This is of particular concern in black patients and other racial groups. 

There is therefore an urgent need to establish more stringent criteria. However, before this is possible, large detailed studies are required to establish the normal range of trabeculation amongst healthy subjects of different ages and ethnicities. Only then can we interpret studies examining trabeculation amongst patients with different forms of cardiomyopathy.

## Figures and Tables

**Table 1:** Echocardiographic criteria for diagnosis of IVNC.

(i) Presence of multiple echocardiographic trabeculations, particularly in the apex and free wall of the left ventricle
(ii) Multiple deep intertrabecular recesses communicating with the ventricular cavity, as demonstrated by colour Doppler imaging
(iii) A twos layered structure of the endomyocardium with a ratio of end-systolic non-compacted endocardial layer to compacted epicardial layer of >2.0 in adults and >1.4 in children
(iv) Absence of other congenital or acquired heart disease, particularly those causing left ventricular outflow obstruction
